# Nature and consequences of interactions between *Salmonella enterica* serovar Dublin and host cells in cattle

**DOI:** 10.1186/s13567-019-0720-5

**Published:** 2019-11-27

**Authors:** Prerna Vohra, Christina Vrettou, Jayne C. Hope, John Hopkins, Mark P. Stevens

**Affiliations:** The Roslin Institute and Royal (Dick) School of Veterinary Studies, University of Edinburgh, Easter Bush, Edinburgh, EH25 9RG UK

## Abstract

*Salmonella enterica* is a veterinary and zoonotic pathogen of global importance. While murine and cell-based models of infection have provided considerable knowledge about the molecular basis of virulence of *Salmonella*, relatively little is known about salmonellosis in naturally-affected large animal hosts such as cattle, which are a reservoir of human salmonellosis. As in humans, *Salmonella* causes bovine disease ranging from self-limiting enteritis to systemic typhoid-like disease and exerts significant economic and welfare costs. Understanding the nature and consequences of *Salmonella* interactions with bovine cells will inform the design of effective vaccines and interventions to control animal and zoonotic infections. In calves challenged orally with *S*. Dublin expressing green fluorescent protein (GFP) we observed that the bacteria were predominantly extracellular in the distal ileal mucosa and within gut-associated lymph nodes 48 h post-infection. Intracellular bacteria, identified by flow cytometry using the GFP signal, were predominantly within MHCII^+^ macrophage-like cells. In contrast to observations from murine models, these *S.* Dublin-infected cells had elevated levels of MHCII and CD40 compared to both uninfected cells from the same tissue and cells from the cognate tissue of uninfected animals. Moreover, no gross changes of the architecture of infected lymph nodes were observed as was described previously in a mouse model. In order to further investigate *Salmonella*-macrophage interactions, net replication of *S. enterica* serovars that differ in virulence in cattle was measured in bovine blood-derived macrophages by enumeration of gentamicin-protected bacteria and fluorescence dilution, but did not correlate with host-specificity.

## Introduction

*Salmonella enterica* subspecies *enterica* is a bacterial pathogen of global importance for humans and animals. The World Health Organisation estimated that *S. enterica* caused 78 million cases of foodborne illness, 59 000 deaths and the loss of 4.1 million disability-adjusted life years during 2010 [1]. Farmed animals are key reservoirs of human non-typhoidal salmonellosis and infections are frequently associated with ingestion or handling of contaminated meat. In the United States, *Salmonella* is endemic in cattle and human infections have been attributed to both beef and dairy cattle [2]. Zoonotic infections are partly a consequence of the ability of *Salmonella* to survive within the bovine lymphatic system and contaminate peripheral lymph nodes, which can enter the food chain via ground beef products [3–8]. Effective vaccines or treatments to limit this are currently lacking. We recently used sequencing-based approaches to study the relative risk of *S. enterica* serovars entering the bovine lymphatic system [3] and to identify *S*. Typhimurium genes required for lymph node colonization [6], but the nature and consequences of interactions at a cellular level in cattle remains relatively poorly understood.

In orally infected cattle, *Salmonella* colonizes the intestines in a manner that requires Type III secretion systems (T3SS)-1 and -2 [9]. Studies using a bovine ligated intestinal loop model established that these systems are also required for the induction of inflammatory and secretory responses [10, 11]. For serovar Dublin, which can cause typhoid-like systemic disease in cattle, we previously observed efficient translocation to the mesenteric lymph nodes draining the distal ileum and dissemination via efferent lymph using a surgical cannulation model [12, 13]. Translocation via efferent lymph occurred in a predominantly cell-free niche and required T3SS-1, but not T3SS-2, at least during the first 24 h after inoculation [13]. In contrast, serovar Gallinarum, which is naturally avirulent in calves by the oral route, was significantly less able to colonize mesenteric lymph nodes (MLN) and spread via efferent lymph, despite being as invasive as *S*. Dublin in bovine ileal loops [12]. The specific cell tropism and consequences for both the host cell and microbe during these infections remain ill-defined beyond a report that MHCII^+^ cells in the lamina propria of the ileal mucosa of calves can contain *Salmonella* [13]. In mouse models, it has been demonstrated that *Salmonella* are carried from the gut to mesenteric lymph nodes by dendritic cells and not macrophages [14–16]. It has also been shown that during systemic infections in mice responses to *Salmonella* lipopolysaccharide (LPS) induce changes to lymph node architecture, which could be a strategy to evade the host adaptive immune responses [17]. Moreover, in vitro infection of human and murine cells with *Salmonella* has been reported to reduce the surface expression of MHCII [18–20], indicating interference with antigen presentation via MHCII that may enable intracellular bacteria to evade immune surveillance. In this study, we aimed to determine whether these observations hold true in cattle by analysing the cell types that are infected by *S.* Dublin in its natural bovine host, measuring the expression of MHCII and co-stimulatory molecules on infected cells and investigating intracellular bacterial net replication following infection of cells ex vivo.

## Materials and methods

### Bacterial strains and culture conditions

A spontaneous nalidixic acid-resistant variant of *Salmonella* Dublin 3246, SD3246 *nal*^*R*^, which is fully virulent in calves [12, 13], was studied here. To aid visualization of infected cells in vivo, SD3246 *nal*^*R*^ was electroporated with the plasmid pFPV25.1, which carries *gfpmut3A* under the control of the *rpsM* promoter resulting in the constitutive synthesis of GFP [21]. This strain (SD3246-GFP) was routinely cultured at 37 °C in Luria–Bertani (LB) broth and on MacConkey agar supplemented with 20 µg mL^−1^ nalidixic acid and ampicillin at 100 µg mL^−1^ to maintain pFPV25.1. Strains of *S*. Typhimurium (ST4/74 *nal*^*R*^) and *S*. Gallinarum (SG9 *nal*^*R*^) have been described previously [12] and are known to induce pathology typical of the wider serovar in animal models. The parent strains used have been fully sequenced [22].

### Isolation of peripheral blood-derived mononuclear cells and culture of macrophages

Animal experiments were conducted according to the requirements of the Animals (Scientific Procedures) Act 1986 (license PPL 60/4420) with the approval of the local Ethical Review Committee. Whole blood was collected from *Salmonella*-free cattle in heparin at a final concentration of 10 U mL^−1^ (Pump-Hep, Leo Pharma, Berkshire, UK) and following dilution with an equal volume of phosphate-buffered saline (PBS), overlaid on 15 mL of Histopaque 1083 (Sigma-Aldrich, Gillingham, Dorset, UK). Following centrifugation at 1200 *g* for 35 min with the brake off, the buffy layer at the interface containing peripheral blood mononuclear cells (PBMCs) was collected and transferred to a new tube. The cells were washed initially at 250 *g* for 10 min followed by 2 washes at 100* g* for 10 min, after which they were resuspended in an appropriate volume of tissue culture medium (RPMI-1640 with GlutaMAX™ and 25 mM HEPES supplemented with 10% foetal bovine serum; FBS) and viable cells were counted. PBMCs were resuspended in RPMI-1640 at 1 × 10^7^ cells mL^−1^ and 75 cm^3^ flasks were seeded with 10 mL of culture. Following incubation for 2 h at 37 °C in 5% CO_2_, non-adherent cells were removed and 20 mL of tissue culture medium (RPMI-1640 medium supplemented with 20% FBS, 4 mM l-glutamine, 0.1% β-mercaptoethanol) was added. PBMCs were incubated for 14 days to allow for differentiation into macrophages with two media changes on days 4 and 10. The macrophages were harvested by removing the tissue culture medium, washing twice with PBS and lifting adherent macrophages using 4 mL of TrypLE Express (Invitrogen, Renfrew, UK). After 10 min of incubation at 37 °C, the macrophages were suspended in fresh medium, centrifuged at 200* g* for 10 min and resuspended for counting. Macrophages were added to 24-well plates such that each well contained 2.5 × 10^5^ cells and incubated overnight at 37 °C in 5% CO_2_ to allow for adherence.

### Validation of pFVP25.1 for detection of *S*. Dublin in vitro using macrophages

An overnight culture of SD3246-GFP was diluted in tissue culture medium to approximately 2.5 × 10^7^ bacterial cells mL^−1^ and added to macrophages in triplicate at a multiplicity of infection (MOI) of 100, which was confirmed retrospectively by plating of serial dilutions to selective agar. Plates were centrifuged at 110 *g* for 5 min and incubated for 3 h at 37 °C in 5% CO_2_. Infected macrophages were harvested as described above and fixed in 4% paraformaldehyde (PFA) in PBS for 15 min, washed in PBS and permeabilised with 0.5% Triton-X 100 for 10 min. Following three washes in 0.1% Tween-PBS, cells were incubated with 1:1500 anti-Group D *Salmonella* LPS (Abd Serotec, Kidlington, UK) for 30 min at 4 °C in the dark. Cells were washed in 0.1% Tween-PBS three times and incubated with 1:500 goat anti-mouse Ig Alexa Fluor 568 (Life Technologies, Renfrew, UK) for 30 min at 4 °C in the dark. Cells were then washed and resuspended in flow cytometry buffer (3% BSA, 0.02% NaN_3_ in PBS) and stored at 4 °C in the dark. Flow cytometry was performed on the LSR II Fortessa (BD, Swindon, UK) acquiring a minimum of 100 000 events using FACSDiva software. The data collected were analysed using FlowJo software version number 8.1.1 (Treestar, Ashland, USA).

### Validation of pFVP25.1 for detection of *S*. Dublin in vivo using a bovine ligated ileal loop model

A 28-day-old Friesian bull calf was confirmed to be culture-negative for *Salmonella* by enrichment of faecal samples in Rappaport–Vassiliadis broth at 37 °C for 18 h followed by plating on MacConkey agar at 37 °C for 24 h. Following this, the calf was anaesthetized with pentobarbital and 6 cm-long intestinal loops were constructed in the mid-ileum using braided surgical silk with 2 cm spacers as described previously [11]. The calf was maintained under general anaesthesia throughout using isofluorane in oxygen. SD3246 *nal*^*R*^ and SD3246-GFP were grown at 37 °C in LB broth with the appropriate antibiotics to give approximately 9 log_10_ colony forming units (CFU) mL^−1^. Ileal loops were injected in triplicate in a semi-randomised order with 5 mL of bacterial culture to study invasion and survival over 10 h and three loops injected with 5 mL of sterile LB broth were maintained as controls. The calf was killed at 10 h post-infection by an overdose of pentobarbital and all loops were excised. Tissue sections from each loop were transported to the laboratory promptly on ice in an isotonic mucosal medium (0.75% choline chloride, 0.27% KCl, 1.8% glucose, 0.5% choline bicarbonate, 1% 10× Minimum Essential Medium Eagle with Earle’s salts, 1% foetal calf serum, 20 mM l-glutamine and 0.3% NaHCO_3_ in distilled water).

The ileal mucosa of each loop was washed gently in PBS to remove non-adherent bacteria from the luminal surface. One gram of each loop, including LB broth-inoculated control loops, was dissociated in 9 mL of PBS using a gentleMACS Dissociator in gentleMACS C Tubes to prepare single cell suspensions. Suspensions were filtered through 70 µm strainers and cells were collected by centrifuging at 500 *g* for 10 min. Cell suspensions were then washed twice in PBS and used to identify infected bovine cells and determine the invasion potential of SD3246-GFP. To identify infected bovine cells, 10^7^ cells from each sample were processed as described above with anti-*Salmonella* LPS. To compare invasion of SD3246 *nal*^*R*^ and SD3246-GFP total and intracellular viable counts of bacteria were determined before and after gentamicin treatment, respectively. For total counts, serial tenfold dilutions were plated in triplicate onto MacConkey agar containing 20 µg mL^−1^ nalidixic acid and incubated overnight at 37 °C, and for intracellular counts, samples were treated with gentamicin at 100 ng µL^−1^ for 30 min at 37 °C to kill extracellular bacteria. Invasion was calculated as a percentage of intracellular bacteria compared to total bacteria in a sample. Control samples from uninfected loops were spiked with SD3246 *nal*^*R*^ and subjected to gentamicin treatment to confirm that the treatment dose and time effectively killed extracellular bacteria.

### Oral challenge of calves with SD3246-GFP and sample collection

Three 28-day-old Friesian bull calves were housed in a secure animal unit and fed on a diet of fresh milk. Calves were confirmed to be culture-negative for *Salmonella* as before. The inoculum for oral challenge was prepared by inoculating several colonies of SD3246-GFP in LB broth with 20 µg mL^−1^ nalidixic acid and 100 µg mL^−1^ ampicillin and incubating statically at 37 °C for 18 h. Twenty milliliter of the bacterial culture was mixed with 20 mL of ant-acid (5% Mg(SiO_3_)_3_, 5% NaHCO_3_, and 5% MgO in sterile distilled water) to promote colonization and administered orally to each calf by syringe before the morning feed. Serial tenfold dilutions of the inoculum were plated in triplicate on MacConkey agar containing 20 µg mL^−1^ nalidixic acid to determine the challenge dose retrospectively. Calves were fed as normal following challenge and were monitored every 12 h as previously described [10]. Post-mortem examinations were performed at 48 h post-infection. Three age- and breed-matched uninfected calves were used as controls and their post-mortem examinations were conducted immediately after confirming that they were *Salmonella*-free.

A section of distal ileal mucosa, mesenteric lymph nodes draining the distal ileal loop and caecal lymph nodes (collectively referred to as LNs) were collected from infected calves and controls. Left and right peripheral lymph nodes (PLNs) including the prescapular, prefemoral and popliteal lymph nodes were also collected. Instruments were changed for each site sampled. Samples were collected in an isotonic mucosal medium as before and promptly transported to the laboratory on ice.

### Bacteriological analysis of tissues from orally inoculated calves

The ileal mucosa was washed gently in PBS to remove non-adherent bacteria from the luminal surface and LNs and PLNs were trimmed of excess fat and fascia before processing. One gram of tissue was homogenized in 9 mL of PBS using a gentleMACS Dissociator in gentleMACS M Tubes. Serial tenfold dilutions were plated in triplicate onto MacConkey agar containing 20 µg mL^−1^ nalidixic acid and incubated overnight at 37 °C. Total and intracellular bacterial counts were determined as before. To confirm the stability of pFPV25.1 in vivo, serial dilutions of gentamicin-protected bacteria from single cell suspensions were plated in triplicate on MacConkey agar containing 20 µg mL^−1^ nalidixic acid with or without ampicillin, which selects for pFPV25.1. The limit of detection for *Salmonella* was 100 CFU g^−1^ of tissue. Tissues that did not yield any colonies were enriched in LB broth with 20 µg mL^−1^ nalidixic acid to confirm presence or absence of *Salmonella*. Tissue homogenates (10^−1^ dilution) from uninfected controls were plated on MacConkey agar without antibiotics to confirm the absence of *Salmonella* and other nalidixic acid resistant bacterial species.

### Immunological analysis of tissues

Single cell suspensions were prepared from the tissues collected using a gentleMACS Dissociator in gentleMACS C Tubes. Ileal mucosa and LN suspensions were prepared in PBS. Suspensions were filtered through 70 µm strainers. Cells were collected by centrifuging at 500 *g* for 10 min and were washed twice in PBS. Cell pellets were re-suspended in PBS and counted. For each sample, 96-well plates were prepared such that each contained 10^7^ cells, which were subjected to multi-parametric staining protocols for flow cytometry. Viability of the cells was determined using the Zombie Violet™ Fixable Viability kit (BioLegend) according to the manufacturer’s instructions. Cells were then stained with primary mouse monoclonal antibodies for 15 min. Cells were stained simultaneously for MHCII DQ (clone CC158, IgG2a, Bio-Rad, Kidlington, UK) and each of the following cell surface molecules: CD40 (IL-A158, IgG1, Bio-Rad), CD80 (IL-A159, IgG1, Bio-Rad), CD86 (IL-A190, IgG1, Bio-Rad), CD11b (IL-A15, IgG1, Institute for Animal Health), CD11c (IL-A16, IgG1, Institute for Animal Health) and CD1w2 (CC20, IgG2a, Institute for Animal Health). Cells were then washed in flow cytometry buffer and centrifuged at 500 *g* for 2 min. Secondary antibodies were added to the cells for 15 min in the dark. The antibodies used were goat anti-mouse IgG1-RPE (Thermo-Fisher, Renfrew, UK) and goat anti-mouse IgG2a-PECy7 (Abcam, Cambridge, UK). Cells were washed in flow cytometry buffer as before and fixed in 2% PFA in PBS for 15 min. After a final wash, the cells were resuspended in flow cytometry buffer and stored at 4 °C in the dark. Flow cytometry was performed on the LSR II Fortessa (BD) acquiring a minimum of 100 000 events using FACSDiva software. The data collected were analysed using FlowJo software version number 8.1.1 (Treestar).

### Microscopy

Blocks of LN tissue from infected and uninfected calves were fixed in 2% PFA in PBS and haematoxylin and eosin (H&E) staining was performed.

### Gentamicin-protection assay

Overnight cultures of SD3246 *nal*^*R*^, ST4/74 *nal*^*R*^ and SG9 *nal*^*R*^ in LB broth with 20 µg mL^−1^ nalidixic acid were diluted in tissue culture medium to approximately 5 × 10^5^ bacterial cells mL^−1^. Tissue culture medium on macrophages was replaced with bacterial cultures, giving a MOI of 2, which was confirmed retrospectively. Plates were centrifuged at 110 *g* for 5 min and incubated for 1 h at 37 °C in 5% CO_2_, following which gentamicin was added to the wells at a final concentration of 100 ng µL^−1^ for 30 min to kill extracellular bacteria. Wells were then washed three times with 1 mL pre-warmed PBS and macrophages were lysed with 1% Triton X-100 in PBS to release intracellular bacteria. Serial tenfold dilutions were plated in triplicate on LB agar with 20 µg mL^−1^ nalidixic acid to determine initial invasion. To assess intracellular survival over time, the medium containing 100 ng µL^−1^ gentamicin was replaced with medium containing 20 ng µL^−1^ gentamicin and the numbers of intracellular bacteria were estimated as before at 4, 6 and 24 h post-infection. A colorimetric assay for release of lactate dehydrogenase was used to confirm an absence of direct cytotoxicity during the assays that could have interfered with the gentamicin-protection assay.

### Fluorescence dilution assay

Cultures of SD3246 *nal*^*R*^, ST4/74 *nal*^*R*^ and SG9 *nal*^*R*^ carrying the plasmid pFCcGi, which expresses mCherry constitutively and has an arabinose-inducible GFP expression system [23], were grown overnight in magnesium minimal medium (Mg-MES; 170 mM 2-(*N*-morpholino) methanesulfonic acid (MES) at pH 5.0, 5 mM KCl, 7.5 mM (NH_4_)_2_SO_4_, 0.5 mM K_2_SO_4_, 1 mM KH_2_PO_4_, 10 mM MgCl_2_, 38 mM glycerol, and 0.1% Casamino Acids) [24], supplemented with 100 µg mL^−1^ ampicillin and 0.2% l-arabinose for induction of GFP production. The following day, cultures were diluted in tissue culture medium to obtain approximately 5 × 10^5^ cells mL^−1^. The assay was performed in the same manner as the gentamicin-protection assay but instead of collecting bacteria for counts, they were collected for analysis by flow cytometry to assess intracellular replication as described previously [23]. Bacteria were collected at 1, 4, 6 and 24 h post-infection, for comparison with the gentamicin-protection assay. They were washed twice in PBS and fixed in 2% PFA in PBS for 15 min. Flow cytometry was performed on the LSR II Fortessa (BD) acquiring a minimum of 10 000 events using FACSDiva software. Fluorescence intensities for GFP and mCherry were detected at 525/50 nm and 610/20 nm, respectively and data were analysed with FlowJo Software version 8.1.1 (Treestar). Replication was measured as the fold change in the geometric mean of GFP fluorescence intensity between the collected cells and the overnight culture as described previously [23].

### Statistical analysis

Statistical tests were performed in GraphPad Prism version 8.00 (GraphPad Software). Data are expressed as mean ± standard deviation (SD) of observations from three calves obtained from three technical replicates. The Wilcoxon signed rank test was used to compare levels of cell surface molecules on GFP^+^ and GFP^−^ cells in tissues of infected animals. The Mann–Whitney test was used to compare levels of cell surface molecules on cells isolated from infected and uninfected animals. Area under the curve analysis followed by ANOVA was used to compare growth and replication of *Salmonella* serovars in macrophages over time. *P* values of ≤ 0.05 were considered to be statistically significant.

## Results

### GFP is a reliable signal to detect *Salmonella*-infected bovine cells in vivo

The constitutive expression of GFP from the *rpsM* promoter of plasmid pFPV25.1 provided a strong and reliable signal for the flow cytometric detection of SD3246-GFP-infected macrophages in vitro (Additional file 1, panels A–C). By contrast, the analysis of infected cells using a rabbit polyclonal anti-*Salmonella* LPS antibody resulted in non-specific signals, which could indicate bacterial debris or non-specific binding of the antibody (Additional file 1, panels A–C). The presence of pFPV25.1 reduced the invasion of macrophages in vitro and of the ileal mucosa in vivo (*P* = 0.0312) (Additional file 1, panels D, E) as previously reported [25]. Nonetheless, total numbers of SD3246 *nal*^*R*^ (mean = 7.5 log_10_ CFU g^−1^) and SD3246-GFP (mean = 7.1 log_10_ CFU g^−1^) recovered from ileal loops were consistent with earlier studies [12]. Moreover, 98.5% of all intracellular bacteria recovered from infected tissues of orally-challenged calves maintained pFPV25.1 without ampicillin selection, confirming both its stability in vivo and that GFP expression can be used to reliably track infected cells (Additional file 1F).

### *S.* Dublin is primarily extracellular in vivo

**Figure 1:**
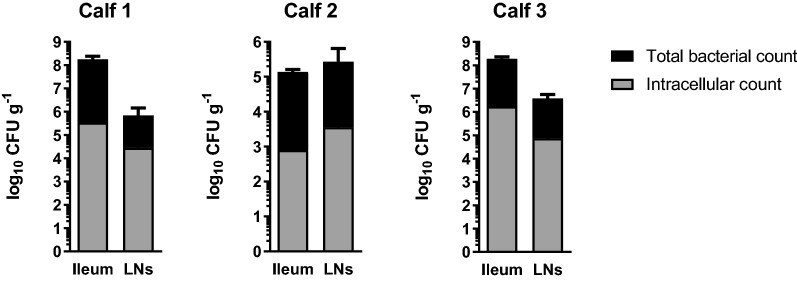
**Location of** ***Salmonella*** **in tissues of orally-challenged cattle.** Three calves were challenged orally with an inoculum containing 10.67 log_10_ CFU SD3246-GFP and viable bacteria were recovered from the ileum and LNs 48 h after infection. The mean total number of SD3246-GFP recovered from the distal ileum and LNs was 7.81 log_10_ CFU g^−1^ and 6.31 log_10_ CFU g^−1^, respectively. Homogenised tissues were also treated with gentamicin to determine the intracellular bacterial counts. The majority of the bacteria were sensitive to gentamicin treatment suggesting that they were extracellular. SD3246-GFP spiked into cell suspensions in vitro in comparable numbers to those found in cell suspensions from tissue was fully eliminated by gentamicin treatment (not shown).

Following oral infection of calves with 10.67 log_10_ CFU of SD3246-GFP, the same clinical signs of pyrexia (Additional file 1G) and diarrhoea from 24 h onwards were observed as previously described for infection with SD3246 *nal*^*R*^ [11, 12]. Large numbers of SD3246-GFP bacteria were recovered from the distal ileum (mean = 7.81 log_10_ CFU g^−1^) and draining mesenteric and caecal LNs (mean = 6.31 log_10_ CFU g^−1^) of the three infected calves (Figure 1). By treating single cell homogenates of tissue with gentamicin, SD3246-GFP was found to be predominantly extracellular in both the ileal mucosa and the LNs at 48 h post-infection; intracellular bacteria accounted for only 0.59 ± 0.34% of the population recovered from the ileal mucosa and 2.46 ± 1.74% of bacteria recovered from the lymph nodes across the three infected calves (Figure 1). No nalidixic acid resistant bacteria were detected in the tissues of uninfected animals, confirming that the bacteria isolated from infected animals were SD3246-GFP.

### *S.* Dublin primarily infects MHCII^+^ macrophage-like cells in ileal mucosa and LNs

**Figure 2:**
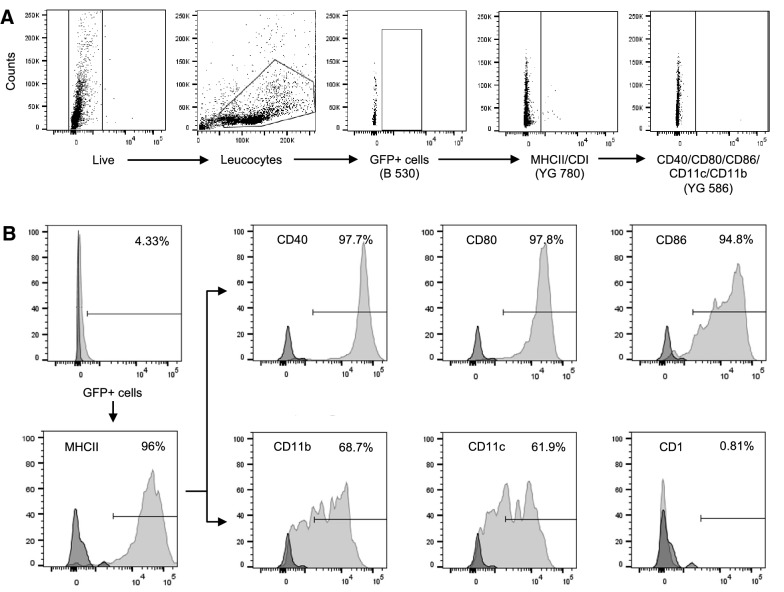
**Identification and characterisation of** ***Salmonella*-infected bovine cells.** Single cell suspensions were prepared from the ileum and LNs of the three calves challenged orally with SD3246-GFP. **A** Viability of the cells was assessed by flow cytometry. Live leucocytes in the samples were selected followed by identification of SD3246-GFP-infected cells using the GFP signal. The majority of infected cells were found to be MHCII^+^. The expression of other cell surface molecules was then studied. **B** The majority of SD3246-GFP-infected cells also expressed CD40, CD80, CD86, CD11b and CD11c but lacked CD1 expression suggesting that they were macrophages. The data shown here are from mesenteric lymph nodes of an infected calf and are representative of infected ileal mucosa and LNs.

SD3246-GFP-infected cells from the ileal mucosa and LNs of challenged calves were identified by the GFP signal. Analysis of the cell surface molecules expressed by these cells indicated that they were predominantly MHCII^+^ (ileum: 92.15 ± 0.99%, LNs: 97.28 ± 2.50%). The GFP^+^ cells also expressed CD40 (ileum: 98.47 ± 1.05%, LNs: 96.77 ± 2.80%), CD80 (ileum: 90.80 ± 6.66%, LNs: 94.87 ± 3.95%), CD86 (ileum: 89.40 ± 5.40%, LNs: 87.40 ± 8.84%), CD11b (ileum: 70.05 ± 7.60%, LNs: 74.27 ± 17.15%) and CD11c (ileum: 69.22 ± 10.20%, LNs: 78.62 ± 10.99%) on their surface and lacked CD1 expression (ileum: 0.24 ± 0.09%, LNs: 0.23 ± 0.13%), suggesting that they were macrophage-like cells. Data are represented as mean ± SD of observations from three calves obtained from three technical replicates. The gating strategy is shown in Figure 2A and representative plots of these infected cells are shown in Figure 2B.

### Effect of *S*. Dublin infection on the expression of cell surface molecules

**Figure 3:**
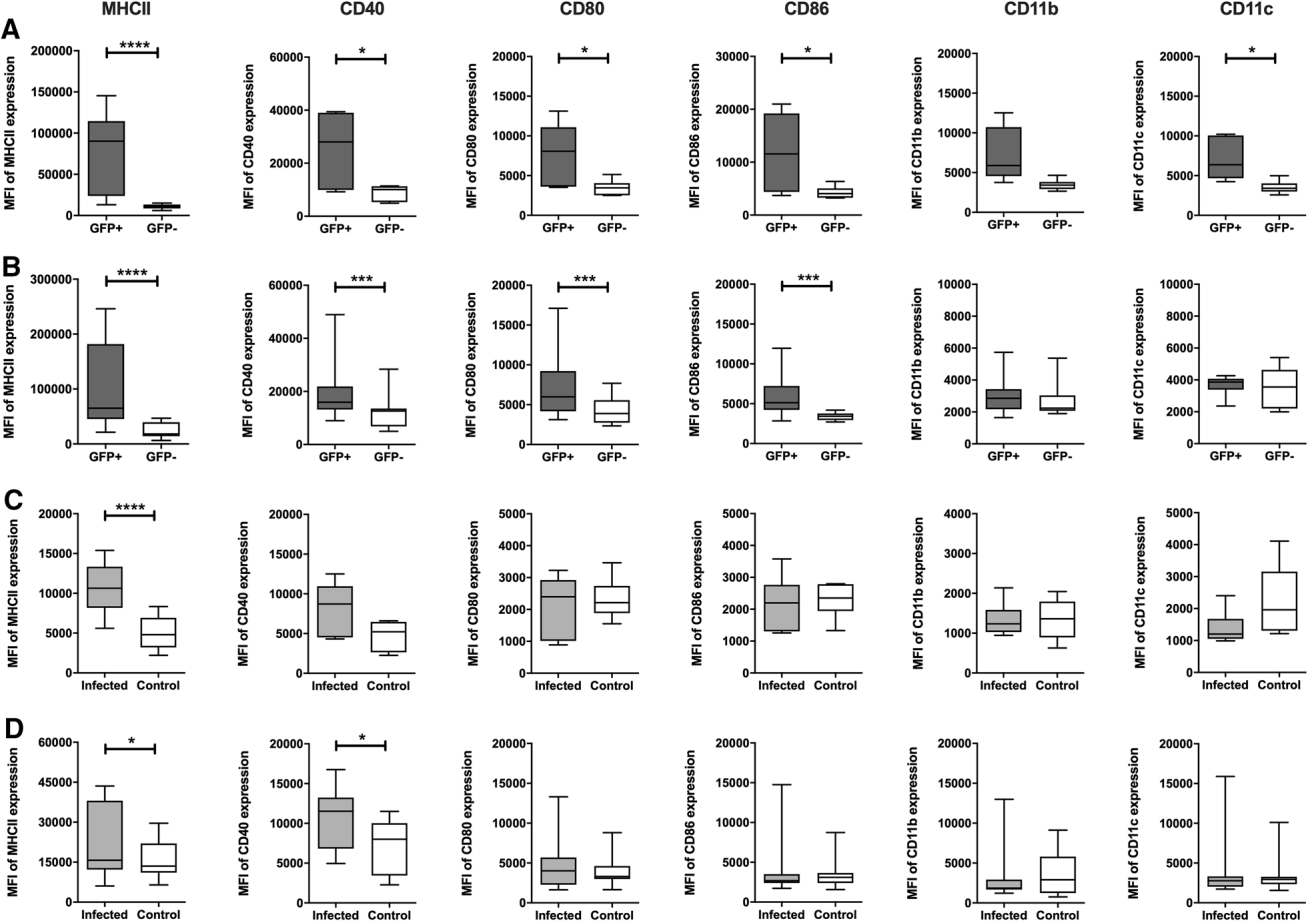
**Effect of** ***Salmonella*** **infection on cell surface molecule expression.** Single cell suspensions prepared from the ileum and LNs of the three calves challenged orally with SD3246-GFP were analysed for expression of several cell surface molecules. SD3246/GFP-infected cells in the **A** ileum and **B** LNs of infected calves showed increased surface expression of MHCII compared to uninfected cells in the same tissue as well as greater expression of CD40, CD80, CD86, CD11b and CD11c. At a tissue level only expression of MHCII and CD40 differed significantly between cells recovered from the **C** ileum and **D** LNs of infected and uninfected control calves. Low levels of CD80, CD86, CD11b and CD11c were detected in the tissues of both infected and uninfected calves and were not significantly different. *P < 0.05, ****< 0.001 and ****P < 0.0001.

SD3246-GFP-infected cells in the distal ileum and LNs expressed significantly higher levels of MHCII on their surface when compared to non-infected GFP^−^ cells from the same tissue (Figure 3). Infected cells also showed significantly greater surface expression of CD40, CD80, CD86 and CD11c. CD11b expression was also higher on infected cells but a significant difference was only observed in the LNs of infected calves. Further, MHCII and CD40 expression in the ileum and LNs of infected calves was significantly higher than the expression of these molecules in the same tissues in uninfected *Salmonella*-free calves of the same age and breed (Figure 3).

### Survival of *S*. Dublin within bovine macrophages

**Figure 4:**
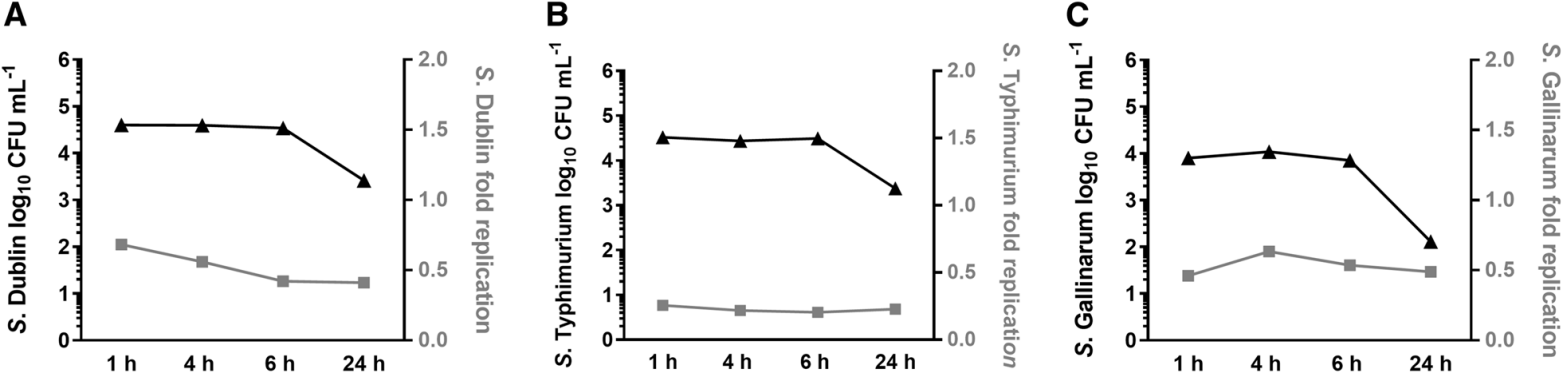
**Survival and replication of** ***Salmonella*** **within infected cells.** Following the identification of macrophages as the primary cell type infected by SD3246-GFP in tissues of orally-challenged calves, the survival and replication of *Salmonella* in peripheral blood-derived macrophages was studied in vitro. Invasion assays were performed in triplicate for *S*. Dublin (**A**), *S*. Typhimurium (**B**) and *S*. Gallinarum (**C**) with gentamicin treatment following an hour of incubation with macrophages and maintenance of gentamicin at a sub-inhibitory concentration thereafter. It was found that all three serovars could survive within bovine macrophages for up to 24 h (black line), with a drop in viability between 6 and 24 h. To study the replication of the serovars macrophages were similarly infected with strains containing the fluorescence dilution plasmid pFCcGi. It was observed that the rate of replication of intracellular *S*. Dublin (**A**), *S*. Typhimurium (**B**) and *S*. Gallinarum (**C**) declined slightly over the first 6 h of infection and then plateaued until 24 h despite differences in their in vivo phenotype and disease presentation.

Invasion of peripheral blood-derived macrophages with SD3246 *nal*^*R*^ and enumeration of viable bacteria showed that *S*. Dublin survived at steady levels within infected cells for up to 6 h. A reduction in bacterial counts was only observed at 24 h post-infection. Assessment of the replication rate of SD3246 *nal*^*R*^ within these cells by analysis of fluorescence dilution using plasmid pFCcGi showed that replication reduced slightly over the first 6 h of infection and then ceased between 6 and 24 h (Figure 4). Similar trends of bacterial survival and replication were observed for ST4/74 *nal*^*R*^ and SG9 *nal*^*R*^ (Figure 4), which respectively cause acute enteritis or no pathology when given to calves of the same age and breed at the same dose as SD3246 [12]. No statistically significant differences in the survival or replication of the three strains were detected.

### Effect of *S*. Dublin infection on MLN architecture

**Figure 5:**
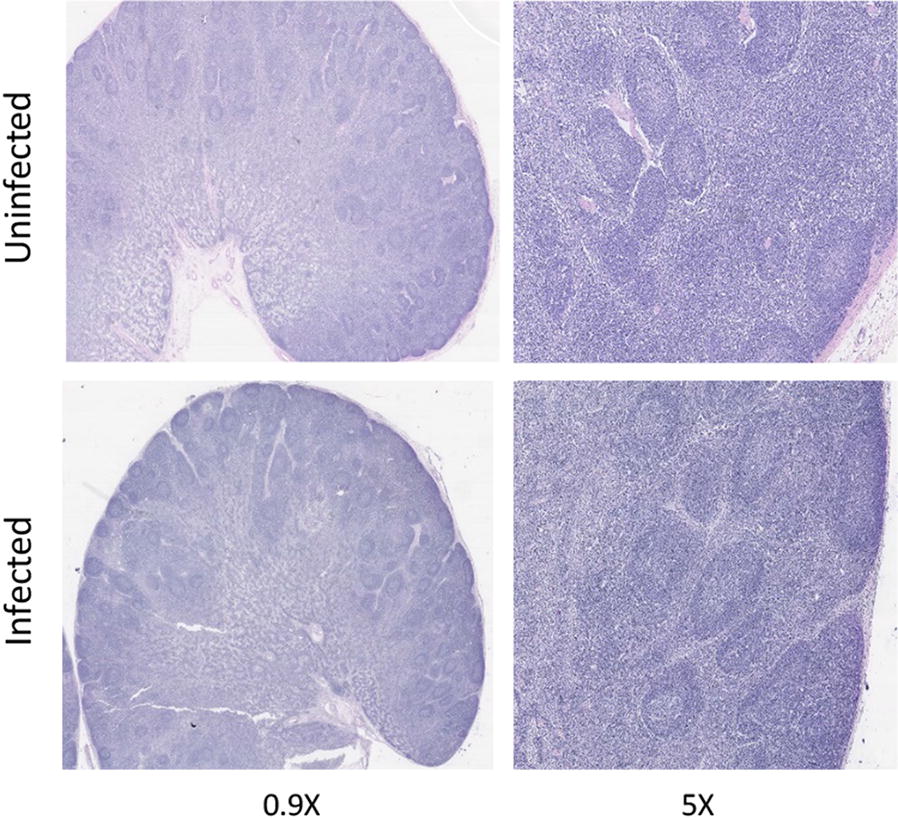
**Effect of** ***Salmonella*** **infection on lymph node architecture.** MLN sections from calves orally challenged with SD3246-GFP and cognate tissues from uninfected calves were H&E stained to assess changes in their gross architecture. No changes in MLN architecture were observed following infection with SD3246-GFP.

Haematoxylin and eosin staining of MLNs archived from infected and uninfected calves showed that the gross architecture of the tissues remained unaltered following infection (Figure 5).

## Discussion

Cattle are a significant reservoir of human non-typhoidal salmonellosis and suffer from salmonellosis in their own right and yet, owing to the challenges of large animal research and a paucity of immunological reagents, much remains unknown about *Salmonella* infections in this species. Here, we sought to understand the nature and consequences of *Salmonella* infections in cattle and to ascertain whether knowledge accrued from murine and cell-based models of infection holds true for *S*. Dublin in cattle or if host- or serovar-specific differences exist.

Infection of cattle with GFP-expressing *Salmonella* Dublin (SD3246-GFP) resulted in the expected clinical signs of infection for wild-type *S*. Dublin [12, 13, 26] (Additional file 1G). As expected, large numbers of bacteria were detected in the gut and associated lymph nodes but they were predominantly gentamicin-sensitive and thus inferred to be extracellular. *Salmonella* has been previously been reported to translocate via efferent lymph in a cell-free niche in cattle [13] and more recently, in a murine model of infection [27], but the presence of large numbers of extracellular bacteria in the bovine gut mucosa and lymph nodes has not been reported before.

In contrast to LPS staining, the GFP signal from SD3246-GFP was highly specific and reliably separated infected bovine cells from uninfected cells in the same tissue. Characterisation of viable infected cells using cell surface molecules confirmed them to be macrophage-like cells. The infected bovine cells expressed MHCII, CD40, CD80, CD86, CD11b and CD11c but did not express CD1, a cell surface molecule used to identify bovine monocyte-derived dendritic cells (DCs). This is similar to observations in sheep where *S. enterica* serovar Abortusovis was mainly associated with granulocytes and macrophages but rarely with DCs [28]. It is also consistent with a report that found SD3246 in lamina propria MHCII^+^ cells by confocal microscopy of bovine ileal mucosa [13], but the present study adds greater resolution in terms of the proportions of cells infected and their expression of co-stimulatory molecules. In response to infection, bovine macrophages significantly increased the expression of MHCII and all the other molecules assessed relative to uninfected cells in the same samples as well as cognate tissues from uninfected animals, and this observation was consistently observed in both distal ileal mucosa and the draining mesenteric and caecal lymph nodes. While similar analyses were applied to the peripheral lymph nodes, the relatively low number of infected cells at these sites, as observed previously [3], did not allow for *Salmonella*-host cell interactions to be accurately defined in the prefemoral, prescapular and popliteal lymph nodes sampled.

The increase in MHCII expression on SD3426-GFP-infected cells in vivo is contrary to previous findings, for example, it has been reported that bovine peripheral blood-derived macrophages infected with a low dose of *Salmonella* Typhimurium did not show any increase in MHCII expression 24 h post-infection but peripheral blood-derived DCs did [29]. It is possible that this was a consequence of the source of the macrophages, which were blood-derived rather than the macrophage-like cells that were infected in situ in the gut and lymph nodes of the cattle here, which were infected by a natural route and sampled at a time when expected pathology was observed. The different *Salmonella* serovar studied could also account for the differences in macrophage responses. In vitro infections of MelJuSo cells, HeLa-CIITA cells and murine bone marrow-derived DCs have also been shown to reduce cell surface expression of MHCII post-infection, and hypothesised to interfere with induction of adaptive responses as a consequence [18–20]. However, these observations could also be serovar- and cell type-specific and do not reflect in vivo observations in SD3246-GFP-infected cattle.

Some *Salmonella* serovars are capable of surviving within lymph nodes [3] and it has been suggested that the host immune response to *Salmonella* LPS via TLR4 results in disruption of lymph node architecture 24 h after infection aiding the evasion of adaptive immune responses [17]. However, in this study a footpad model of infection was used to study *Salmonella* interactions in the popliteal lymph node. In calves infected with SD3246-GFP via the natural route, no such gross architectural changes were observed in the draining mesenteric lymph nodes or caecal lymph nodes 48 h after oral challenge compared to uninfected control animals.

An observation following infection of bovine cells that is consistent with previous work from *Salmonella*-infected mice and murine cells is that *Salmonella* can persist in a non-replicative but viable state within host macrophages [30]. The lack of replication of *S*. Dublin in bovine peripheral blood-derived macrophages beyond 6 h post-inoculation was confirmed by a fluorescence dilution method [23, 30] and survival up to 24 h was confirmed by gentamicin-protection assays. These tests were extended to *S*. Typhimurium, a serovar with a broad host range which causes acute enteritis in cattle, and *S*. Gallinarum, an avian-specific serovar which is avirulent in cattle but still capable of gut invasion [12]. It was found that strains of these serovars enter and persist within bovine macrophages at a comparable level to *S*. Dublin. The observation that net replication of serovars in bovine blood-derived macrophages does not correlate with their virulence in calves is consistent with earlier work showing that replication of serovars Typhimurium, Choleraesuis and Dublin in porcine primary alveolar macrophages is not correlated with host-specificity of these strains in orally dosed pigs [31].

In summary, we have identified bovine macrophages as the cell type primarily infected by *S*. Dublin in the ileum and lymph nodes during bovine salmonellosis and studied the effects of this infection on cell surface molecule expression and have also studied the replicative state and survival of the bacteria in macrophages ex vivo. Several of the observations in this study contradict previously published observations from murine models and in vitro infection studies, which could be owing to the model hosts and cells used as well as the *Salmonella* serovar studied. This highlights the importance of studying salmonellosis in a naturally-affected target host species. Moreover, as enteric salmonellosis in cattle closely resembles that in humans, [32] observations made during typhoidal infections of cattle as studied here may also be relevant to human disease and may offer greater insight than murine models of salmonellosis.

## Supplementary information

**Additional file 1. Validation of pFPV25.1 for detection of** ***Salmonella*-infected bovine cells.** Several in vitro and in vivo tests were performed to assess the suitability of pFPV25.1 for detection of *Salmonella*-infected cells. (A–C) Firstly, bovine peripheral blood-derived macrophages were infected in vitro with SD3246-GFP at a MOI of 100 and infected cells were identified using either the constitutive GFP-expression from pFPV25.1 or by staining with anti-*Salmonella* LPS. (A) While the majority of GFP^+^ (infected) cells were also LPS^+^, a proportion of infected cells were incorrectly identified by anti-LPS staining. (B) The GFP signal from infected cells was sensitive and specific. (C) Detection of infected cells by anti-LPS staining was less specific than GFP. (D) Following this, the effect of presence of pFPV25.1 on bacterial invasion of peripheral blood-derived macrophages was determined in vitro and it was found that SD3246-GFP was less invasive than wild-typec SD3246 *nal*^*R*^. (E) This phenotype was confirmed in vivo using the bovine ligated ileal loop model. While SD3246-GFP was less invasive than SD3246 *nal*^*R*^ after 10 h of infection, the total bacterial numbers recovered were similar from loops inoculated with both strains. (F) Following oral challenge of calves with SD3246-GFP, the stability of pFPV25.1 was confirmed to inform the accuracy of identifying *Salmonella*-infected cells. It was found that pFPV25.1 was stably maintained within intracellular bacteria recovered from tissues of these calves 48 h post-infection, even in the absence of ampicillin selection, providing confidence that the GFP signal was used to accurately detect infected bovine cells. (G) Lastly, the effect of presence of pFPV25.1 on clinical signs was assessed and it was found that the pyrexia induced in SD3246-GFP-challenged calves was the same as that expected during wild-type *S*. Dublin infections, confirming that virulence of SD3246-GFP had not been adversely affected.

## Abbreviations

*S*. Dublin, SD3246: *Salmonella* Dublin 3246
GFP: green fluorescent protein
T3SS: type III secretion system
MLN: mesenteric lymph nodes
LPS: lipopolysaccharide
LB: Luria–Bertani
PFA: paraformaldehyde
PLN: peripheral lymph nodes
PBS: phosphate-buffered saline
LNs: mesenteric and caecal lymph nodes
MOI: multiplicity of infection
MES: methanesulfonic acid
