# Supplementary figures and images for: Nature and consequences of interactions between Salmonella enterica serovar Dublin and host cells in cattle
Source: Vet Res. 2019 Nov 27;50:99. doi: 10.1186/s13567-019-0720-5 (PMC6880441; doi:10.1186/s13567-019-0720-5)

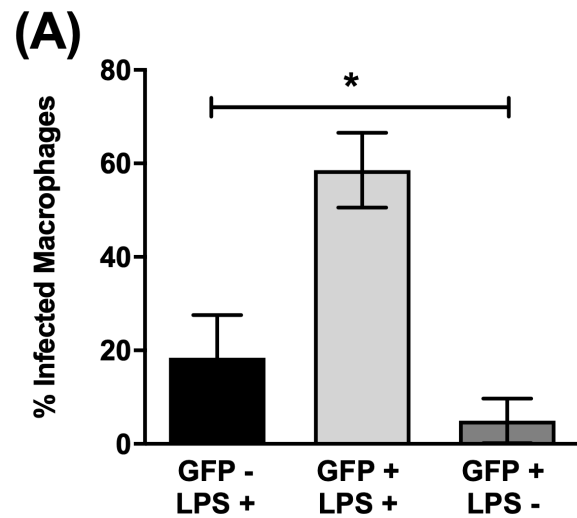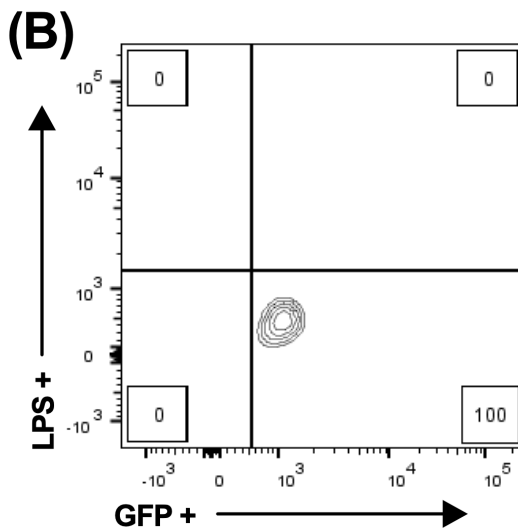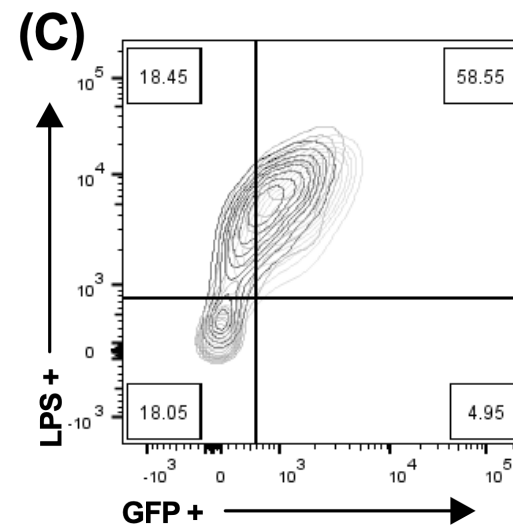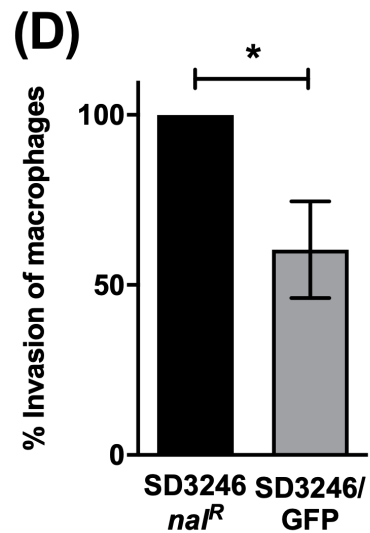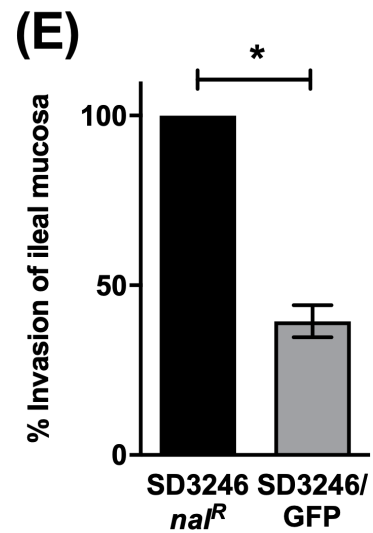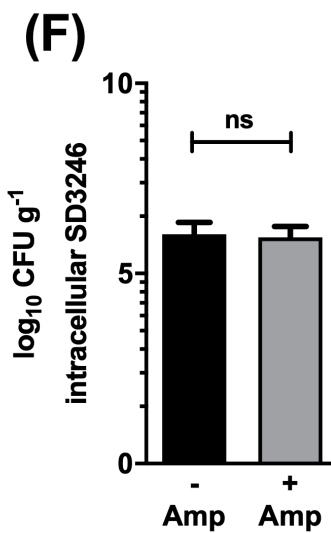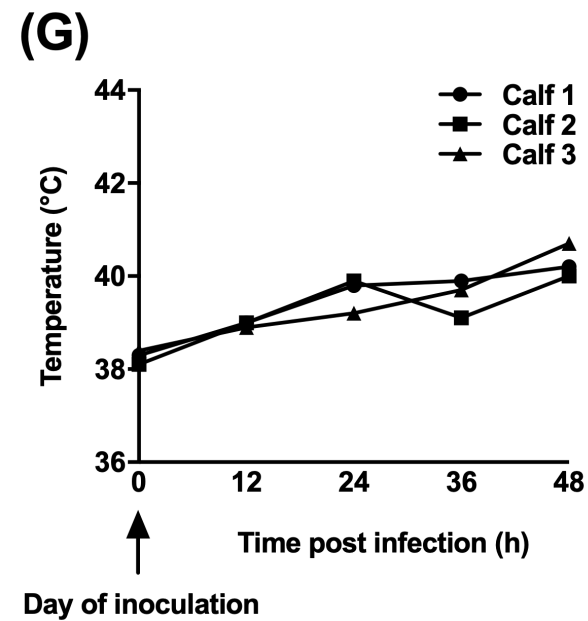

Supplement: Supplementary file 1 — Additional file 1. Validation of pFPV25.1 for detection of Salmonella-infected bovine cells. Several in vitro and in vivo tests were performed to assess the suitability of pFPV25.1 for detection of Salmonella-infected cells. (A–C) Firstly, bovine peripheral blood-derived macrophages were infected in vitro with SD3246-GFP at a MOI of 100 and infected cells were identified using either the constitutive GFP-expression from pFPV25.1 or by staining with anti-Salmonella LPS. (A) While the majority of GFP+ (infected) cells were also LPS+, a proportion of infected cells were incorrectly identified by anti-LPS staining. (B) The GFP signal from infected cells was sensitive and specific. (C) Detection of infected cells by anti-LPS staining was less specific than GFP. (D) Following this, the effect of presence of pFPV25.1 on bacterial invasion of peripheral blood-derived macrophages was determined in vitro and it was found that SD3246-GFP was less invasive than wild-typec SD3246 nalR. (E) This phenotype was confirmed in vivo using the bovine ligated ileal loop model. While SD3246-GFP was less invasive than SD3246 nalR after 10 h of infection, the total bacterial numbers recovered were similar from loops inoculated with both strains. (F) Following oral challenge of calves with SD3246-GFP, the stability of pFPV25.1 was confirmed to inform the accuracy of identifying Salmonella-infected cells. It was found that pFPV25.1 was stably maintained within intracellular bacteria recovered from tissues of these calves 48 h post-infection, even in the absence of ampicillin selection, providing confidence that the GFP signal was used to accurately detect infected bovine cells. (G) Lastly, the effect of presence of pFPV25.1 on clinical signs was assessed and it was found that the pyrexia induced in SD3246-GFP-challenged calves was the same as that expected during wild-type S. Dublin infections, confirming that virulence of SD3246-GFP had not been adversely affected. [file 13567_2019_720_MOESM1_ESM.pdf]
